# Grb2 monomer–dimer equilibrium determines normal versus oncogenic function

**DOI:** 10.1038/ncomms8354

**Published:** 2015-06-24

**Authors:** Zamal Ahmed, Zahra Timsah, Kin M. Suen, Nathan P. Cook, Gilbert R. Lee, Chi-Chuan Lin, Mihai Gagea, Angel A. Marti, John E. Ladbury

**Affiliations:** 1 Department of Biochemistry and Molecular Biology, University of Texas, M.D. Anderson Cancer Center, Unit 1000, 1515 Holcombe Boulevard, Houston, Texas 77030, USA; 2 Center for Biomolecular Structure and Function, University of Texas, M.D. Anderson Cancer Center, Unit 1000, 1515 Holcombe Boulevard, Houston, Texas 77030, USA; 3 School of Molecular and Cellular Biology, University of Leeds, Leeds LS2 9JT, UK; 4 Department of Chemistry, Rice University, Houston, Texas 77005, USA; 5 Department of Veterinary Medicine and Surgery, University of Texas, M.D. Anderson Cancer Center, Unit 63, 1515 Holcombe Boulevard, Houston, Texas 77030, USA

## Abstract

The adaptor protein growth factor receptor-bound protein 2 (Grb2) is ubiquitously expressed in eukaryotic cells and involved in a multitude of intracellular protein interactions. Grb2 plays a pivotal role in tyrosine kinase-mediated signal transduction including linking receptor tyrosine kinases to the Ras/mitogen-activated protein (MAP) kinase pathway, which is implicated in oncogenic outcome. Grb2 exists in a constitutive equilibrium between monomeric and dimeric states. Here we show that only monomeric Grb2 is capable of binding to SOS and upregulating MAP kinase signalling and that the dimeric state is inhibitory to this process. Phosphorylation of tyrosine 160 (Y160) on Grb2, or binding of a tyrosylphosphate-containing ligand to the SH2 domain of Grb2, results in dimer dissociation. Phosphorylation of Y160 on Grb2 is readily detectable in the malignant forms of human prostate, colon and breast cancers. The self-association/dissociation of Grb2 represents a switch that regulates MAP kinase activity and hence controls cancer progression.

## Abstract (web summary)

Grb2 is an adaptor protein that can exist as a dimer that dissociates on phosphorylation of Y160. Here, the authors show that only the monomeric protein is capable of activating mitogen-activated protein kinase signal transduction and hence control oncogenic outcome.

## Graphical abstract

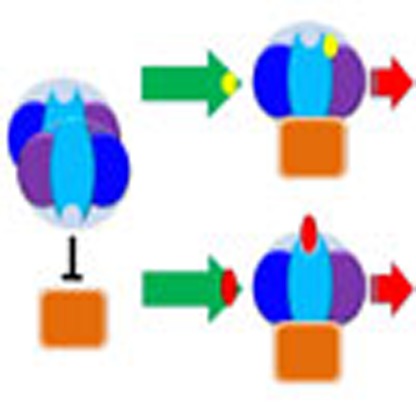

Growth factor receptor-bound protein 2 (Grb2) is an adaptor protein with no intrinsic enzymatic activity which is ubiquitously expressed in all eukaryotic cells. It was originally discovered as epidermal growth factor receptor (EGFR) interacting protein^1^ but is now known to form complexes with a wide variety of cellular proteins including, but not limited to, protein tyrosine kinases, receptor tyrosine kinases, phosphatases, adaptors and intracellular scaffolds^2^,^3^,^4^,^5^,^6^,^7^. Grb2 has a modular architecture consisting of a central Src homology 2 (SH2) domain flanked by two Src homology 3 (SH3) domains. The N-terminal SH3 (N-SH3) has been reported to be constitutively associated with the mammalian homologue of the drosophila protein son-of-sevenless (SOS), a guanine nucleotide exchange factor (GEF) that acts on the Ras subfamily of small GTPases^8^. Under non-stimulatory conditions the Grb2–SOS complex resides in the cytoplasm. However, following receptor activation phosphorylated receptor tyrosine kinases (RTKs) and RTK-docking proteins such as Shc, IRS1-4 and FRS2/3 serve as recognition sites for the Grb2-SH2 domain^6^,^9^. As a result SOS is recruited to the plasma membrane leading to the activation of mitogen-activated protein kinase (MAPK) signalling cascade^10^,^11^.

Mechanisms through which control of signal transduction is exerted are vital in the regulation of cell response and function as well as in disease. With this in mind, two important features of Grb2 function have been reported, but their functional roles have not been fully elucidated: (1) Grb2 exhibits a monomer–dimer equilibrium^12^,^13^,^14^ and (2) tyrosine residues on Grb2 can be phosphorylated (for example by CD3 and CD4 (ref. 15), prolactin^16^ and vascular endothelial growth factor^17^). Furthermore, Grb2 is specifically phosphorylated on tyrosine residue 160 (Y160) by c-Src^18^, BCR-Abl^19^, fibroblast growth factor receptor 2 (FGFR2)^20^, NPM-ALK, TPR-Met and TEL-JAK2 (ref. 21).

Here we resolve these elusive roles by demonstrating that Grb2 is only functional in MAPK signal transduction when it is in its monomeric form; and that dissociation of the constitutive dimer is facilitated by either phosphorylation of Y160 (pY160) or through the binding of a tyrosylphosphate-containing ligand. Therefore, rather than being a passive adaptor, Grb2 functions as a key control point in MAPK signalling. Our data also reveal the presence of high levels of pY160, the monomeric signalling active form of Grb2, in cancer tissue cells and suggest that this could be a marker for proliferative MAPK activation and resulting oncogenic outcome.

## Results

### Grb2 dimer is perturbed by phosphorylation of Y160

**Figure 1:**
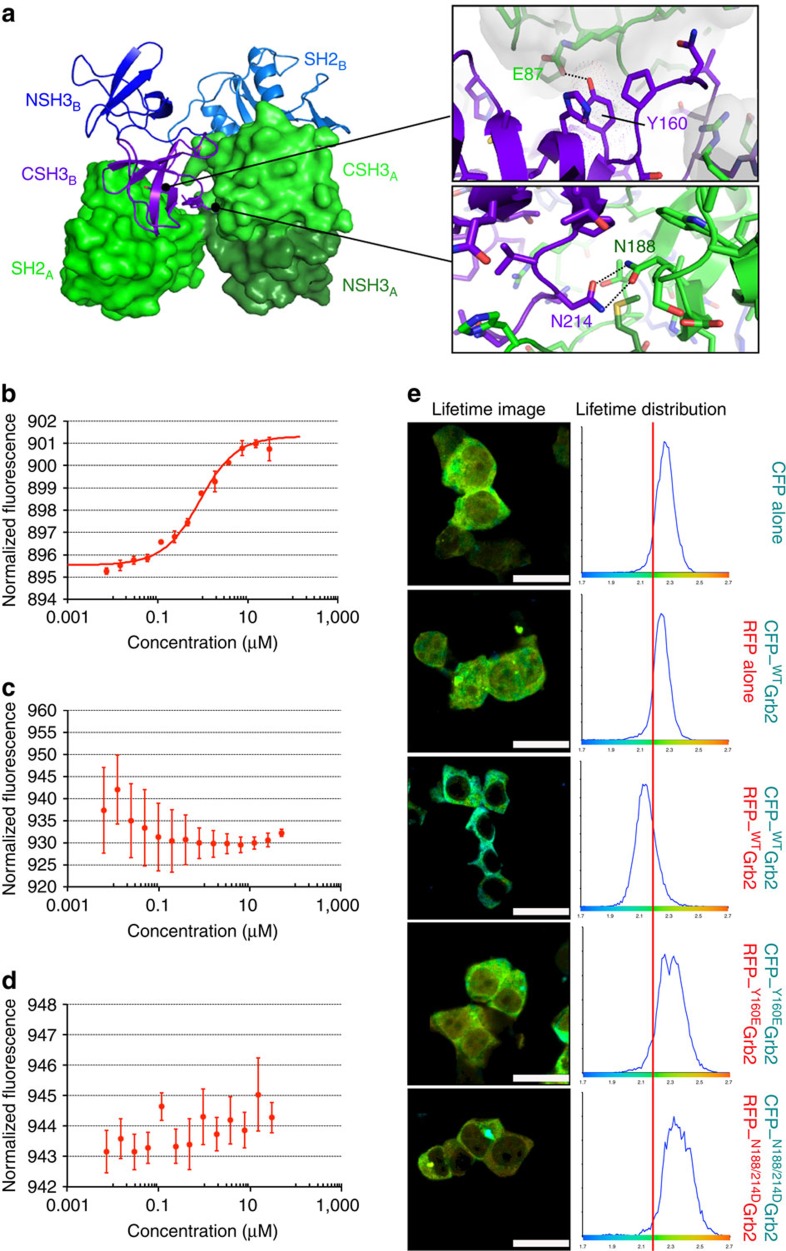
*In vitro* and *in vivo*^WT^Grb2 form dimers. (**a**) Homodimer with individual Grb2 protomers of the dimer depicted using surface (green) and ribbon (blue/purple) representations (derived from PDB 1GRI). Individual SH2 and SH3 domains of the protomers are differentially coloured and labelled. Zoomed box (top)—Y160 of one protomer (purple) is buried in the dimer interface, packing against the CSH3 domain and hydrogen bonding with E87 of the other protomer chain (green). Zoomed box (bottom)—hydrogen bonded Asn188 (green) and Asn214 (purple) in the dimer interface. (**b**) Grb2 dimerization measured by MST. Unlabelled ^WT^Grb2 protein (7.3 nM to 30 μM) was titrated into a fixed concentration of labelled Grb2 (100 nM). The data for thermophoresis was recorded at 20 °C using the blue LED at 20% and IR-Laser at 40%. The isotherm derived from the raw data and fitted according to the law of mass action to yield an apparent *K*_d_ dimer of 0.76±0.20 μM. (**c**) The data points representing the MST results of the Y160E mutant, which show a scattered distribution and unsuitable for fitting. (**d**) The thermophoresis results of the N188/214D mutant, as with Y160E the data is unsuitable for fitting analysis. (**e**) FLIM of Grb2 dimerization in mammalian cells through FRET measurements. CFP- and RFP-tagged wild-type or the Grb2 mutants together with controls were co-transfected in HEK293T cells as indicated on the figure. The results show a clear shortening of the average lifetime distribution for fluorophore in cells expressing the ^WT^Grb2. This is due to FRET between the CFP–Grb2 and RFP–Grb2. The Y160E and N188/214D mutants on the other hand show an average lifetime distribution comparable to the controls indicating no FRET. Scale bar, 25 μm.

We sought to identify the factors regulating Grb2 monomer/dimer equilibrium which might define a switching mechanism for Grb2-mediated signal transduction. Since Y160 lies within the previously reported dimer interface and forms a hydrogen bond with E87 on the partner protomer^13^ (Fig. 1a), tyrosine kinase post-translational modification of Y160 could perturb dimerization through a repulsive charge or steric clash effect.

We generated a phosphorylation charge mimetic Grb2 mutant Y160E, designed such that the charge on the mutated glutamic acid residue repels the proximal E87 in the partner protomer and hence abrogates dimer formation (Fig. 1a). We also produced a mutant whereby two asparagine residues in the Grb2 dimer interface at positions 188 and 214 were mutated to aspartic acids (N188/214D). This mutant was unable to dimerize due to the juxtaposition of the two substituted negatively charged groups (Fig.1a). The N188/214D mutant provides a phosphorylation-independent dimerization-defective control protein to assess the physiological relevance of Y160 phosphorylation. The wild-type Grb2 (^WT^Grb2), Y160E (^Y160E^Grb2) and N188/214D (^N188/214D^Grb2) mutants were expressed and purified from bacteria. To confirm their stability we performed binding studies with known ligands (Supplementary Fig. 1). The monomer–dimer equilibrium was measured by microscale thermophoresis (MST) after N-terminal labelling with Ato488. ^WT^Grb2 has an apparent dimerization constant (*K*_d_) of ∼0.7 μM (Fig.1b) consistent with the previously reported value^12^. The ^Y160E^Grb2 and ^N188/214D^Grb2 on the other hand showed no monomer–dimer transitions (Fig. 1c,d). In order to confirm the monomeric status of the two mutants we performed additional experiments using dynamic light scattering, analytical gel filtration, fluorescence anisotropy and time-resolved fluorescence anisotropy, native polyacrylamide gel electrophoresis (Supplementary Fig. 2). Under all conditions ^WT^Grb2 formed a concentration-dependent dimer whereas the two mutants were incapable of dimer formation.

Dimerization of ^WT^Grb2, ^Y160E^Grb2 and ^N188/214D^Grb2 was assessed in cells using fluorescence lifetime imaging microscopy (FLIM) to measure fluorescence resonance energy transfer (FRET) between two Grb2 protomers. Cyan fluorescence protein (CFP)-tagged Grb2 (CFP–Grb2) and red fluorescence protein (RFP)-tagged Grb2 (RFP–Grb2) were co-transfected into mammalian cells. Grb2 dimerization can only be seen in cells expressing the wild-type Grb2 (CFP–^WT^Grb2/RFP–^WT^Grb2) (Fig. 1e and Supplementary Fig. 3). Cells expressing ^Y160E^Grb2 and ^N188/214D^Grb2 show no evidence of dimerization.

**Figure 2:**
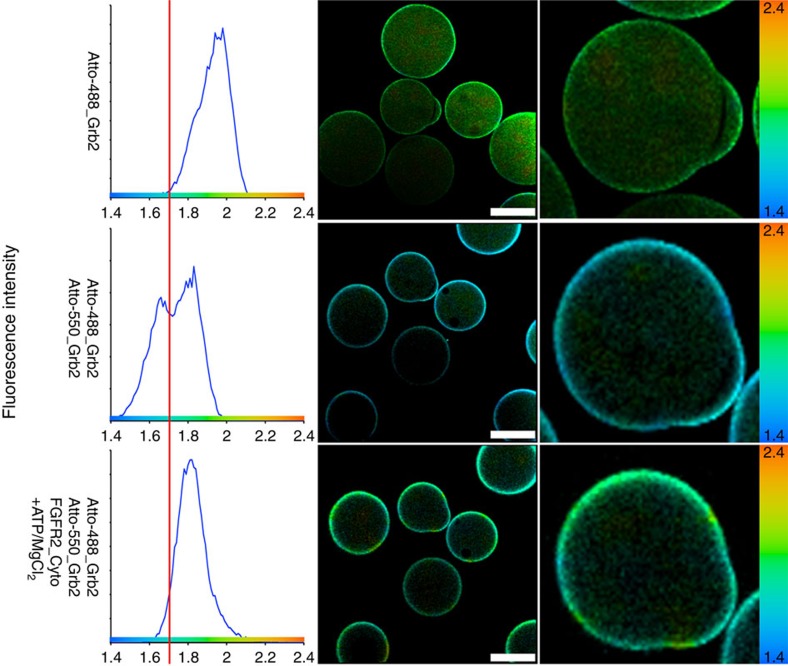
Grb2 tyrosine phosphorylation disrupts dimerization. FRET between Atto488–Grb2 and Atto550–Grb2 measured with FLIM. The panels on the right show the distribution of the average fluorescence lifetime. The middle image is the representation of the measured lifetime as a false colour map. On the right hand side are zoomed images of a bead with a characteristic imperfection. The mean lifetime of Atto488–Grb2 is centred at ∼1.9 ns. The addition of the acceptor Atto550–Grb2 leads to an apparent left shift in the average lifetime. This is clear from the appearance of a new peak at ∼1.6 ns as a result of FRET between Atto488–Grb2 and Atto550–Grb2. Addition of FGFR2 has no effect on the apparent average lifetime. However, further addition of ATP/MgCl_2_ reversed the average lifetime to 1.9 ns providing evidence for the disruption of FRET and hence the disruption of dimerization on phosphorylation of Grb2. Scale bars, 50 μm.

To demonstrate that phosphorylation of the wild-type Grb2 disrupts dimerization, we devised a pseudo single molecule FLIM (smFLIM) experiment. Bacterially expressed and purified His-tagged ^WT^Grb2 was N-terminally labelled with Atto488 and immobilized on Talon beads, while non-tagged ^WT^Grb2 was labelled with Atto550. After measurement of the reference lifetime of Atto488–^WT^Grb2, Atto550–^WT^Grb2 was added and allowed to form a complex. The fluorescence lifetime population showed a distinct left shift to a shorter value indicating that FRET has occurred (Fig. 2). Incubation for one hour in the presence of purified FGFR2 cytoplasmic domain alone had no effect on the FRET lifetime between the Grb2 protomers. However a further hour of incubation in the presence of ATP and MgCl_2_ in order to phosphorylate Y160 on Grb2 (ref. 20) resulted in a shift of the average fluorescence lifetime towards the original reference lifetime for Atto488–^WT^Grb2 alone (Fig. 2), demonstrating that the formation of the Grb2 dimer was abrogated by phosphorylation. These findings clearly demonstrate that the disruption of Atto488–Grb2/Atto550–Grb2 complex is FGFR2 activation dependent.

### Tyrosylphosphorylated ligand results in dimer dissociation

**Figure 3:**
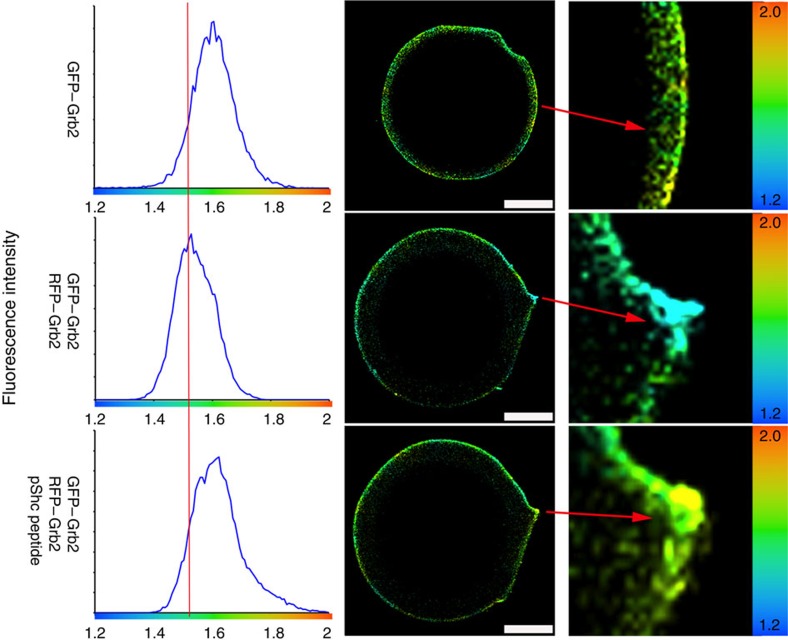
Binding of phosphotyrosine by Grb2-SH2 domain leads to dissociation. smFLIM shows Grb2 dimerization is disrupted upon phospho-Shc peptide binding by its SH2 domain. GFP-tagged Grb2 immobilized on GFP-trap beads were imaged in 20 mM Tris-HCl, 50 mM NaCl at pH 8.0. The reference average lifetime in the absence of acceptor is 1.6 ns. RFP–Grb2 was added and allowed to form complex for 1 h and the lifetime of GFP–Grb2 shortens as indicated by left-shifted peak with average lifetime centred on 1.5 ns clearly indicating that FRET has occurred. Phospho-Shc peptide (10 μM) containing the pYxN motif was then added, allowed to equilibrate for an hour and the fluorescence lifetime was measured. Addition of the phospho-Shc peptide restores the lifetime to the control values obtained in the absence of acceptor. This clearly shows that the phospho-Shc peptide disrupts dimerization of Grb2. The zoomed image of the bead (right hand column) shows lifetime values mapped to a false colour image. The change in fluorescence lifetime as a function of colour is highly noticeable. The data presented here was consistently reproduced in three independent experiments. Scale bars, 10 μm.

From the reported crystal structure^13^ Y160 appears to form a hydrogen bond with E87 of the partner protomer (Fig. 1a). Since E87 lies within the conserved FLI/VRE motif of the SH2 domain (which is involved in recognition of phosphorylated tyrosine residues), the insertion of a tyrosylphosphate into the binding pocket on the SH2 domain might be expected to perturb the side chain position of E87 and disrupt the interaction between Y160 and E87. Using the previously described smFLIM method we determined that the addition of a tyrosylphosphopeptide based on the sequence of the known Grb2 ligand Shc (pShc) leads to dissociation of Grb2 dimers into the monomeric form (mGrb2) (Fig. 3). The addition of a proline-rich peptide derived from SOS which binds to the SH3 domains of Grb2 had no effect on dimerization on the surface of the Talon beads (Supplementary Fig. 4a). We confirmed this observation by measuring the monomer–dimer equilibrium of Grb2 using MST. Since Grb2 is monomeric, in the presence of a saturating concentration of pShc no apparent change in the oligomeric state of the protein was observed (Supplementary Fig. 4b). As expected similar experiments in the presence of the SOS1 peptide, Grb2 undergoes a dimer-to-monomer dissociation which has an identical apparent *K*_d_ (∼0.7 μM) to wild-type protein in the absence of peptide binding (Supplementary Fig. 4c). These results are consistent with previously reported NMR data where Grb2 was found to be monomeric in the presence of a phosphopeptide^22^.

### Monomeric Grb2 is the signalling active form

**Figure 4:**
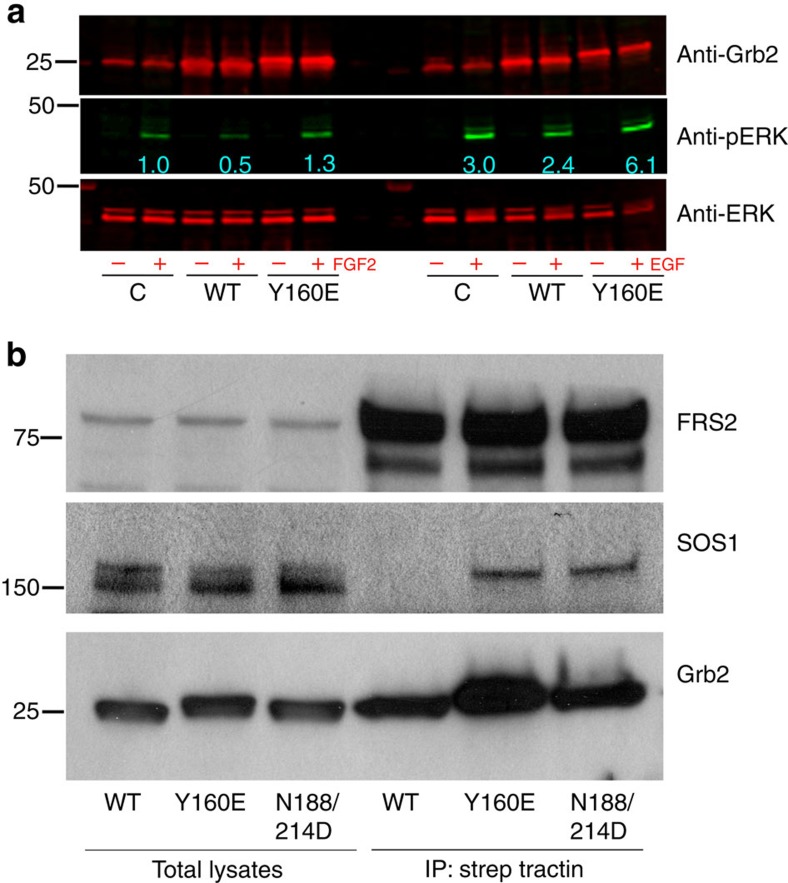
Dimeric Grb2 inhibits while monomeric Grb2 promotes MAP kinase activity. (**a**) Stable HEK293T cells overexpressing FGFR2–GFP (as control, C) and strep-tagged wild-type Grb2 or the dimerization-defective Y160E mutant were stimulated with 50 ng/ml FGF2 or EGF for 15 and 5 min, respectively. Cell-lysates were analysed for phospho-ERK (pERK), total ERK and Grb2 expression levels using specific antibody and Odyssey infra-red imaging. (**b**) HEK293T cells overexpressing FGFR2–GFP with indicated strep-tagged Grb2 in serum were lysed and subjected to strep-tactin affinity purification. The resulting co-precipitated complexes along with input cell-lysates were analysed for SOS and FRS2 binding using respective antibody. The immunoblot was also re-probed for Grb2 as a loading control. The data presented are representative of three independent experiments.

We have shown two independent mechanisms of Grb2 dissociation, both dependent on tyrosine kinase activity. Both these mechanisms could play a fundamental role in Grb2-mediated intracellular signalling. Since Grb2 is known to bind to its downstream effector SOS1, we postulate that only mGrb2 elicits downstream effector function and hence the dimeric Grb2 (dGrb2) is incapable of signal transduction. We tested this hypothesis by investigating the downstream signalling effects of mGrb2 versus dGrb2 in mammalian cells. In cells overexpressing high concentrations the population of Grb2 is expected to be predominantly dGrb2, whilst the dimerization-defective Grb2 mutants will be monomeric regardless of their intracellular concentrations. Cells were transfected with ^WT^Grb2 and ^Y160E^Grb2, and downstream MAPK signalling was compared. The results show that overexpression of ^WT^Grb2 leads to a significant decrease in FGF2- and EGF-induced extracellular signal-regulated kinases (ERK) phosphorylation. An equivalent level of dimerization-defective mutant overexpression on the other hand retains ligand-induced ERK activity (Fig. 4a and Supplementary Fig. 5a). We also confirmed Grb2 concentration-dependent MAPK inhibition by serially increasing in the intracellular Grb2 protein concentration in cells. A direct correlation between increased Grb2 concentration and decreased MAPK was observed (Supplementary Fig. 5c–e). Since the formation of the Grb2–SOS complex is pivotal for RTK-stimulated Ras/MAP kinase activation, we investigated whether the differential complex formation by dGrb2 versus mGrb2 with SOS1 could explain the observed suppression of ERK phosphorylation. Grb2 was affinity purified from cells and the resulting co-precipitating complex was analysed for SOS1 (Fig. 4b and Supplementary Fig. 5b). The results show that the monomeric ^Y160E^Grb2 and ^N188/214D^Grb2 form stable complexes with SOS1 whereas the dimeric ^WT^Grb2 does not. As a control the immunoblot was re-probed with anti-FRS2 antibody, which showed that both dGrb2 and mGrb2 were equally proficient in precipitating the upstream adaptor protein required for FGF receptor signalling.

The direct interaction of either dGrb2 or mGrb2 with SOS1 in cells was tested using FLIM. RFP-tagged ^WT^Grb2 and ^Y160E^Grb2 were co-transfected with GFP-tagged SOS1 (GFP–SOS1). The results show that the monomeric Grb2 mutants form more stable interactions with SOS1 than ^WT^Grb2 (Supplementary Fig. 6). Together these results confirm that a monomeric Grb2 is necessary and sufficient to form the interaction with SOS1 and propagate the MAPK signalling pathway.

### Monomeric Grb2 is associated with malignant cancers

**Figure 5:**
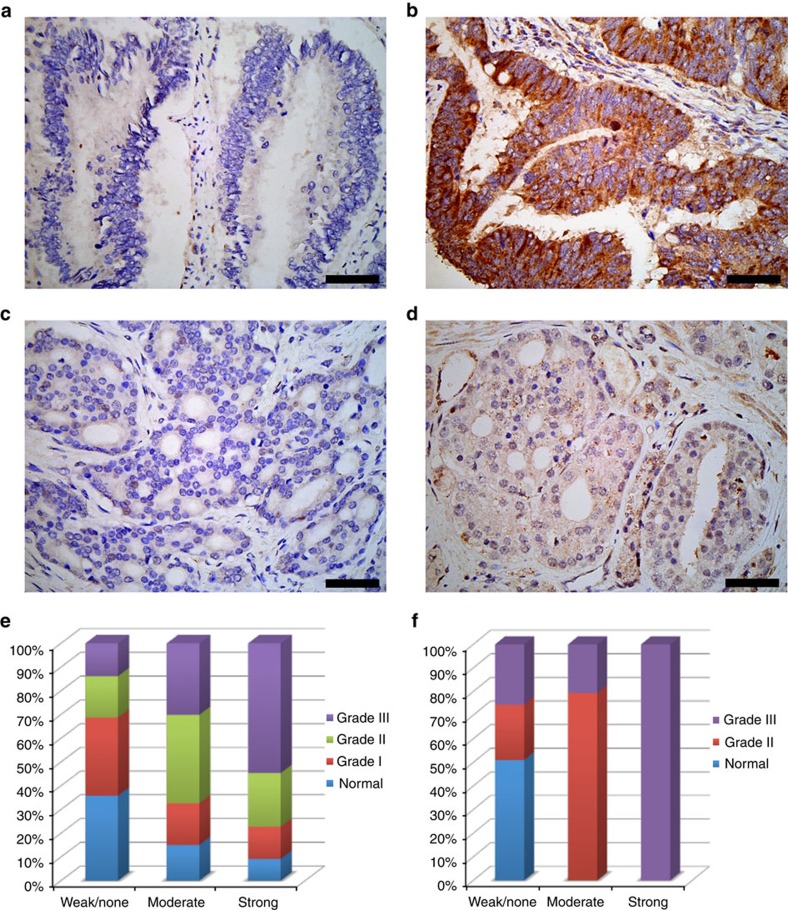
Analysis of human colon and prostate cancer tissues for Y160 phosphorylation. Using the pY160 antibody IHC was performed on multi-tumours tissue microarray of 95 samples with 40 types of tumours from 27 organs. (**a**) Weak or no staining for Grb2 Y160 phosphorylation in grade I colon adenocarcinoma. (**b**) Strong staining for Grb2 Y160 phosphorylation in grade III colon adenocarcinoma. (**c**) Weak or no staining for Grb2 Y160 phosphorylation in grade II prostate adenocarcinoma. (**d**) Moderate staining for Grb2 Y160 phosphorylation in grade III prostate adenocarcinoma. A significant increase in the level of pY160 phosphorylation is seen in higher-grade tumour samples. The bars on **a–d** correspond to 50 μm. (**e**) The pY160 antibody staining patterns for 118 colon cancer tissue samples. The samples were scored according to the pY160 staining as weak or none, moderate and strong and plotted against tumour grade as percentage. Data compiled from normal tissues (*n*=42), and tumours grade I (*n*=28), grade II (*n*=34) and grade III (*n*=14) which shows a progressive increase in the strength of pY160 staining with higher tumour grade. (**f**) The pY160 antibody staining patterns for 42 clinical prostate cancer tissue samples with the relative staining patterns for pY160 antibody. As above, samples were scored, sorted and plotted against tumour grade as percentage. Normal and/or hyperplasic tissue (*n*=15), tumour grade II (*n*=13) and grade III (*n*=14). Here the pY160 staining is only associated with malignant tumours and intensity of staining is increased in tumours with a higher level of malignancy.

Y160 is a target phosphorylation site for many receptor and non-receptor tyrosine kinases that are involved in cancer growth and metastasis^21^. This suggests dGrb2 may have an inhibitory role whilst mGrb2 promotes proliferative downstream signalling. Here we have shown that Y160 phosphorylation-dependent dissociation of dGrb2 is sufficient to induce upregulation of MAPK signalling (Fig. 4) which is associated with proliferation and metastasis^23^,^24^,^25^. Therefore, we would predict that in tyrosine kinase-driven cancers phosphorylation of Y160 and the resulting dissociation of Grb2 dimers would be capable of inducing cell proliferative and metastatic outcome. This would suggest that pY160 could be used as marker for elevated tyrosine kinase-mediated ERK signalling in human cancers. We therefore generated a site-specific anti-pY160 antibody against pY160. This antibody was tested exhaustively and found to be suitable for detecting Y160-phosphorylated Grb2 in western blotting and in immunohistochemistry (IHC) analysis (Supplementary Fig. 7). We performed IHC analysis of a multi-tumour tissue array containing 40 human tumour samples from 27 different organs. The results show that Y160 was highly phosphorylated in a significant number of high-grade malignant tumours but not in the lower grade or non-malignant counterpart tumours or in corresponding normal tissue (Supplementary Fig. 8). Y160 phosphorylation was particularly prevalent in higher grade, late-stage malignant tumours of the colon and prostate (Fig. 5a–d). Therefore, we performed additional IHC analysis on 118 colon and 42 prostate cancer tissues with the anti-pY160 antibody, which shows a clear correlation between disease progression and Grb2 phosphorylation (Fig. 5e,f). Higher levels of Y160 phosphorylation were again clearly associated with higher-grade malignant tumours. In addition we performed protein microarray analysis on 55 human breast tumours with matched normal tissue using the pY160 antibody. The results revealed Y160 phosphorylation was absent in normal breast samples, however 12 out of 55 breast tumour samples were positive for pY160 (Supplementary Fig. 9a–c). To confirm the results of the protein microarray, we selected two positive breast tumour samples and the corresponding normal matched sample for western blotting analysis. The results show elevated Grb2 expression and phosphorylation, MAP kinase and tyrosine kinase activity in the tumour samples compared with the normal cohort. Furthermore, it was possible to assign the elevated pY160 level of one patient to increased EGFR expression and kinase activity (Supplementary Fig. 9d).

## Discussion

The discovery of the adaptor protein Grb2 as receptor tyrosine kinase-interacting protein 20 years ago was a key point in defining RTK signal transduction^26^,^27^. Since then a plethora of Grb2-interacting proteins have been reported (http://string-db.org). The interaction between Grb2 and SOS and the recruitment of the Grb2–SOS complex to the plasma membrane to activate the MAPK protein kinase cascade is a pivotal function of Grb2 in cells. It has been known for some time that Grb2 could form dimers, however it has been debated as to whether the dimeric state had any physiological relevance. Here we used both *in vitro* and *in vivo* studies to show concentration-dependent Grb2 dimerization (Fig. 1 and Supplementary Fig. 2). We have demonstrated that the monomer–dimer population can be perturbed by Y160 phosphorylation and the binding of a ligand to the SH2 domain of Grb2 (Figs 2, 3). We addressed the issue of whether there is a functional consequence of disruption of the dimeric state in cells and demonstrated that the regulation of the self-association state of Grb2 is intimately related to the receptor tyrosine kinase activity. The monomeric form is the signalling-competent active form whereas dimeric Grb2 is the inactive inhibitory form. Only monomeric Grb2 associates with SOS, while both monomeric and dimeric form can engage a phosphorylated tyrosine residue (Fig. 4b).

The change from dimeric–monomeric state in cells is strongly influenced by upregulation of kinase activity and the resultant phosphorylation of Grb2 itself or of cognate ligands. The requirement of Grb2 to dissociate from the dimeric state provides a further ‘check-point' in MAPK signal transduction. Driving the dissociation of Grb2 through increased kinase activity can be reversed by phosphatases. We have previously reported that the tyrosine phosphatase Shp2 is a specific phosphatase for Grb2 (ref. 20).

Phosphorylation, however, is not the only mechanism for dGrb2 dissociation. Protein expression levels will also impact on this. This is reflected in downstream signalling, where overexpression of wild-type Grb2 increases the population of dimer and hence suppresses MAPK activation (Fig. 4a and Supplementary Fig. 5). However, overexpression of Grb2 coupled with Y160 phosphorylation leads to increased MAPK activation (Supplementary Fig. 9). Thus, initiation of MAPK signalling is modulated by several cellular conditions and fluctuations in these could have significant impact on cellular outcome.

Human cancer tissue analysis shows a significant increase in Grb2 phosphorylation in high-grade pre-metastatic tumours, further highlighting the importance of mGrb2 in signal transduction and cancer progression. Therefore, the mechanistic insight gained from this work might pave the way for developing novel strategies towards cancer therapy, based on stabilizing the formation of Grb2 dimers. One inventive way of achieving this might be based on a small molecule which could irreversibly crosslink the two monomers across the dimer interface. Together these results from human cancer patients confirm the importance of mGrb2 in intracellular signalling and suggest that pY160 could be used as a potential predictive biomarker in personalized cancer therapy.

## Methods

### Reagents

Anti-Grb2 (C23), anti-FRS2 and anti-SOS antibodies were obtained from Santa Cruz Biotechnology. Anti-phospho-Erk and anti-total Erk antibodies were from Cell Signaling. All antibodies were diluted to 1:1,000. Anti-pY160 antibody was raised against QPT(pY)VQALFDFDPQC peptide (GenScript). Phospho-EGFR peptide FLPVPE(pY)INQSVPKR, phospho-Shc peptide EEPPDHQ(pY)YN-DFPGK and SOS1 peptide PVPPPVPPRRRPEY were purchased from GenScript. The SomaPlex Protein Microarray of Human Breast Tumor & Normal Tissue (PMA2-001-L), as well as the matching cell lysates were purchased from Protein Biotechnologies. Multi-organ tissue microarray slides (MUT951), colon cancer tissue array (COC1262) and prostate cancer tissue array (PRC481) were purchased from Pantomics.

### Molecular cloning

Gene fragments encoding Grb2 were cloned into pECFP-C1 (Clontech) in the BamHI site to create the CFP–Grb2 construct. The monomeric RFP (mRFP) was cloned into the NheI/HindIII site of the pcDNA3.1(+) vector (Invitrogen). Grb2 was then subcloned into the BamHI/EcoRI sites to create the RFP–Grb2 construct. In both cases the fluorescent tag was placed at the N-terminus of Grb2. For histidine-tagged protein expression and purification, Grb2 was cloned into BamHI/EcoRI site of a modified pET28a vector containing tobacco edge virus protease (TEV) cleavage site. The Y160E and N188/214D mutations were introduced into Grb2 by site-directed mutagenesis. Bacterial protein expression and purification were performed as described previously^20^,^28^,^29^. Strep-tagged Grb2 was created by fusing a WSHPQFEK sequence at the N-terminus of the full-length Grb2. The PCR amplified DNA fragment was inserted into the HindIII/PmeI site of pcDNA6-myc/His C vector (Invitrogen). Bacterial GFP–Grb2 and RFP–Grb2 were created by cloning NdeI–SacI–BamHI-(GFP or RFP)–HindIII–EcoRI–XhoI fragments from pEGFP-N1 or mRFP-pcDNA3.1, respectively, into the NdeI and XhoI site of a pET28a vector. The full-length Grb2 was then subcloned into the HindII/XhoI sites. Molecular cloning expression and purification of FGFR2 cytoplasmic domain in bacteria is described elsewhere^12^.

### Cell culture

HEK293T cells obtained from the American Type Culture Collection were maintained in Dulbecco's modified Eagle's high glucose medium (DMEM) supplemented with 10% (vol/vol) fetal bovine serum (FBS) and 1% antibiotic/antimycotic (Lonza) in a humidified incubator with 10% CO_2_ as described previously^30^,^31^,^32^. Stable HEK293T cells overexpressing GFP-tagged FGFR2 were produced as described previously^30^. Control and Grb2 knockdown cells were produced as described previously^20^. Mammalian cell transfection was performed in a six-well plate using Metafectin Pro (Biontx-USA) reagents according to the manufacturer's instructions. Cells were lysed in 50 mM HEPES (pH 7.5), 1% (v/v) Igepal-C630, 1 mg ml^−1^ bacitracin, 1 mM EDTA, 10 mM NaF, 1 mM sodium orthovanadate, 10% (v/v) glycerol, 50 mM NaCl, 1 mM PMSF and Protease Inhibitor Cocktail Set III (Calbiochem).

### Immunoblotting

Immunoblotting was performed using the indicated antibodies. Blots were cropped such that at least one marker position was present. Uncropped full scans are shown in Supplementary Fig. 10.

### Protein expression and purification

6 × Histidine-tagged constructs of FGFR2 and Grb2 were expressed and purified from *E. coli* as described in refs 28, 32. It should be noted that purified Grb2 is inherently unstable as a monomer and can be affected by inter-molecular disulphide bond formation.

### Cell imaging and FLIM

Cells were co-transfected with fluorescent protein-tagged Grb2 and then grown on glass coverslips for 24 h before experimentation. As controls, cells expressing CFP alone or CFP–^WT^Grb2 with RFP-alone were also analysed. All experiments were carried out in the presence of serum unless indicated. Cells were fixed by addition of 4% (w/vol) paraformaldehyde, pH 8.0. Cells were washed six or seven times with PBS, pH 8.0, and then mounted onto a slide with mounting medium (0.1% p-phenylenediamine/75% glycerol in PBS at pH 7.5–8.0). Cells were imaged using a Leica SP5 II confocal microscope with internal PMT FLIM detector. CFP was excited at 860 nm with titanium–sapphire pumped laser (Mai Tai BB, Spectral Physics) and SPC830 data and image acquisition card for time-correlated single photon counting. Data processing and analysis were done using B&H SPC FLIM analysis software. The fluorescence decays were fit with a single exponential decay model. FLIM experiments were performed as described previously^20^,^28^,^29^,^33^.

### Single molecule FLIM

For Grb2 as FRET donor histidine-tagged Grb2 was bacterially expressed and purified, labelled with Atto488 NHS ester according to the manufacturers' instructions. For Grb2 as FRET acceptor, histidine-tagged Grb2 was expressed and purified and the tag was removed by TEV cleavage. Uncleaved proteins and TEV were removed by subtractive gel filtration. Purified Grb2 was then labelled with Atto 550 NHS ester according to the manufacturer instructions. In both cases free dyes were removed by size exclusion chromatography and quantified. Only labelled proteins with 1:1 (protein:dye) ratio was used for experiments.

Atto488–Grb2 was loaded onto Talon beads and placed on a 35-mm glass-bottom dish (MatTek Corporation) containing 250 μl PBS pH 7.5. Beads were allowed to settle and then the reference lifetime of the Atto488–Grb2 in the absence of acceptor was measured. Without disturbing the beads a final concentration 0.5 μM Atto550–Grb2 was then added and allowed to equilibrate and form dimers for 30–60 min. The lifetime was again measured and proceeded to the next treatment. For each treatment extreme care was taken to avoid disturbing the beads. For smFLIM with GFP-tagged Grb2, GFP–Grb2, was immobilized on GFP-trap agarose beads (Chromotek).

### Immunohistochemistry staining

All tissues utilized in this study were of human origin and purchased from Pantomics Inc and employed in IHC experiments and scoring according to research protocols approved by the IACUC of The University of Texas MD Anderson Cancer Center (Houston, TX). Slides were baked at 60 °C for 30 min and then the temperature was increased to 72 °C. Slides were then rinsed three times with Bond Dewax followed by absolute alcohol and Bond Wash. The temperature was then increased to 100 °C, and Bond ER Solution 1 (pH 6) was applied and incubated for 30 min. Slides were rinsed with Bond Wash and the temperature was allowed to come down to room temperature. Anti-phospho-pY160 antibody was applied at 1:50 dilution (0.6 mg ml^−1^ final) and incubated for 15 min at room temperature followed by rinsing with Bond Wash. Post Primary was applied and incubated for 8 min, then slides were again rinsed with Bond Wash. Bond Polymer was applied and incubated for 8 min followed by rinsing with Bond Wash two times and then with distilled water. Peroxide Block was applied and incubated for 5 min, then rinsed with Bond Wash four times and then washed with distilled water. 3,3′-Diaminobenzidine (DAB) was applied and incubated for 10 min then rinsed with distilled water three times. Haematoxylin was applied and incubated for 7 min, then rinsed with Bond Wash. Slides were then removed from Leica Bond III and rinsed with distilled water four times. They were then dehydrated in graded alcohols and xylene, then coverslipped with ClearMount from American MasterTech using Leica CV5030 coverslipper. Preparation of IHC slides for cultured cells, 5 × 150 mm dishes were used per block. Cells were scraped from dishes with a rubber policeman and pelleted by centrifugation at 800 rpm for 5 min. The cell pellet was fixed overnight with 10% formalin and then changed to 70% ethanol before embedding into formalin fixed paraffin embedded (FFPE) blocks.

### Microscale thermophoresis

The MST method has been described in detail elsewhere^34^. The KD, dimer for Grb2 dimers was measured using the Monolith NT.115 from Nanotemper Technologies. Proteins were fluorescently labelled with Atto488 according to the manufacturer's protocol. Labelling efficiency was determined to be 1:1 (protein to dye) by measuring the absorbance at 280 and 488 nm. A solution of unlabelled Grb2 was serially diluted from about 30 μM to 7.3 nM in the presence of 100 nM labelled Grb2. The samples were loaded into silica capillaries (Polymicro Technologies) after incubation at room temperature for 15 min. Measurements were performed at 20 °C in PBS without calcium and magnesium (Lonza) containing 0.01% Tween 20, by using 20% LED power and 40% IR-laser power. Measurements were also carried out on 40 and 60% IR-Laser power for comparison. Data analyses were performed using Nanotemper Analysis software, v.1.2.101.

## Additional information

**How to cite this article:** Ahmed, Z. *et al.* Grb2 monomer–dimer equilibrium determines normal versus oncogenic function. *Nat. Commun.* 6:7354 doi: 10.1038/ncomms8354 (2015).

## Supplementary Material

Supplementary Information Supplementary Figures 1-10, Supplementary Note 1 and Supplementary References
