# Supplementary material for: Grb2 monomer–dimer equilibrium determines normal versus oncogenic function
Source: Nat Commun. 2015 Jun 24;6:7354. doi: 10.1038/ncomms8354 (PMC4491180; doi:10.1038/ncomms8354)
Supplement: Supplementary Information — Supplementary Figures 1-10, Supplementary Note 1 and Supplementary References [file ncomms8354-s1.pdf]

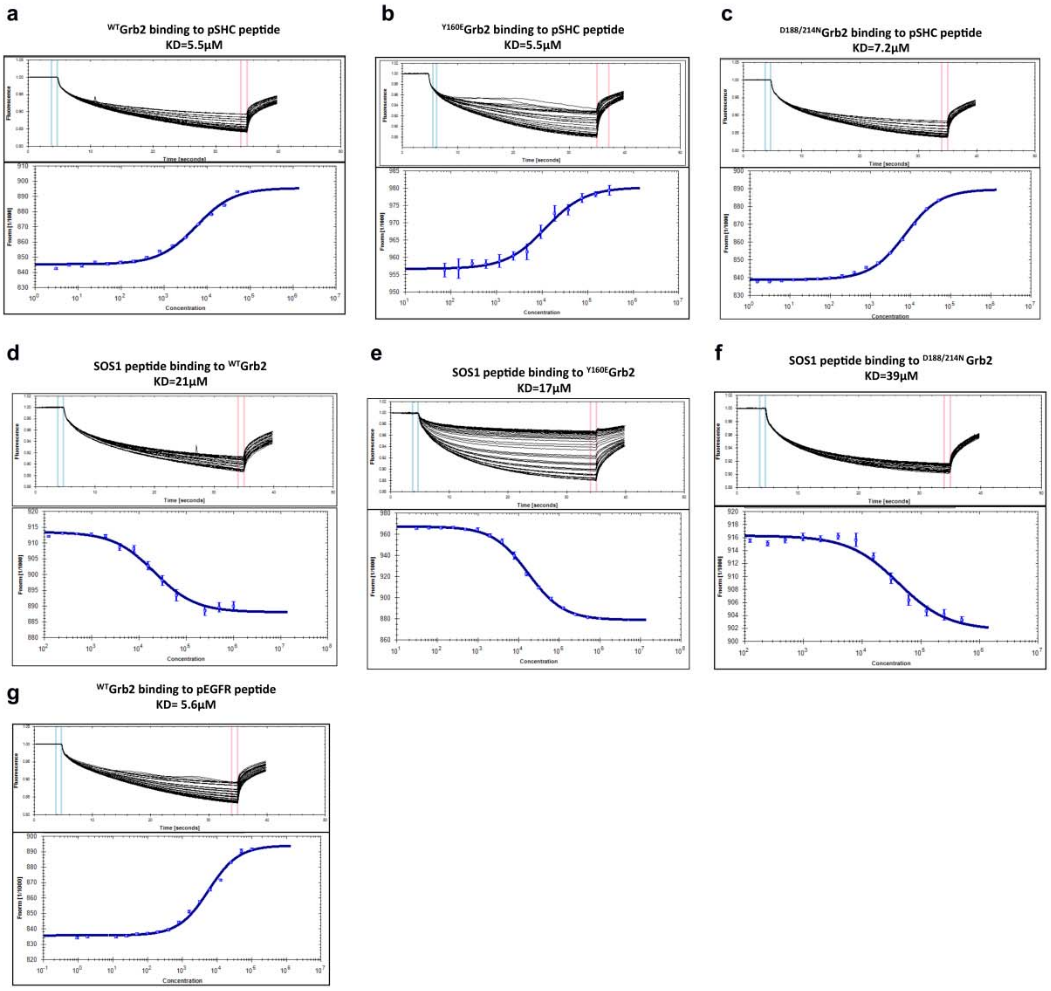

Supplementary Figure 1. Y160E and N188/214D mutant retain pEGFR and SOS peptide binding capacity.

The SH2 and SH3 domain structural integrity and functionality were confirmed by peptide binding assays. Binding studies using two phosphotyrosine peptides derived from EGFR (FLPVPE{pY}IN-QSVPKR) and Shc (EEPPDHQ{pY}YNDFPGK) for the Grb2 SH2 domain and a SOS1-derived proline-rich peptide (PVPPPVPPRRRPEY) for the SH3 domains were performed using MST[1]. Grb2 was fluorescently labeled using Atto488 dye. A solution of unlabeled peptide was serially diluted from about 100 µM to 55 nM in the presence of 200 nM labeled domain. **(a, b** and **c)** binding of phospho-Shc peptide to WT, Y160 and N188/214D with comparable binding affinities. (**d, e** and **f)** binding of SOS1 peptide to WT, Y160 and N188/214D with comparable binding affinities. **g)** Wild type Grb2 binding to phospho-EGFR peptide.

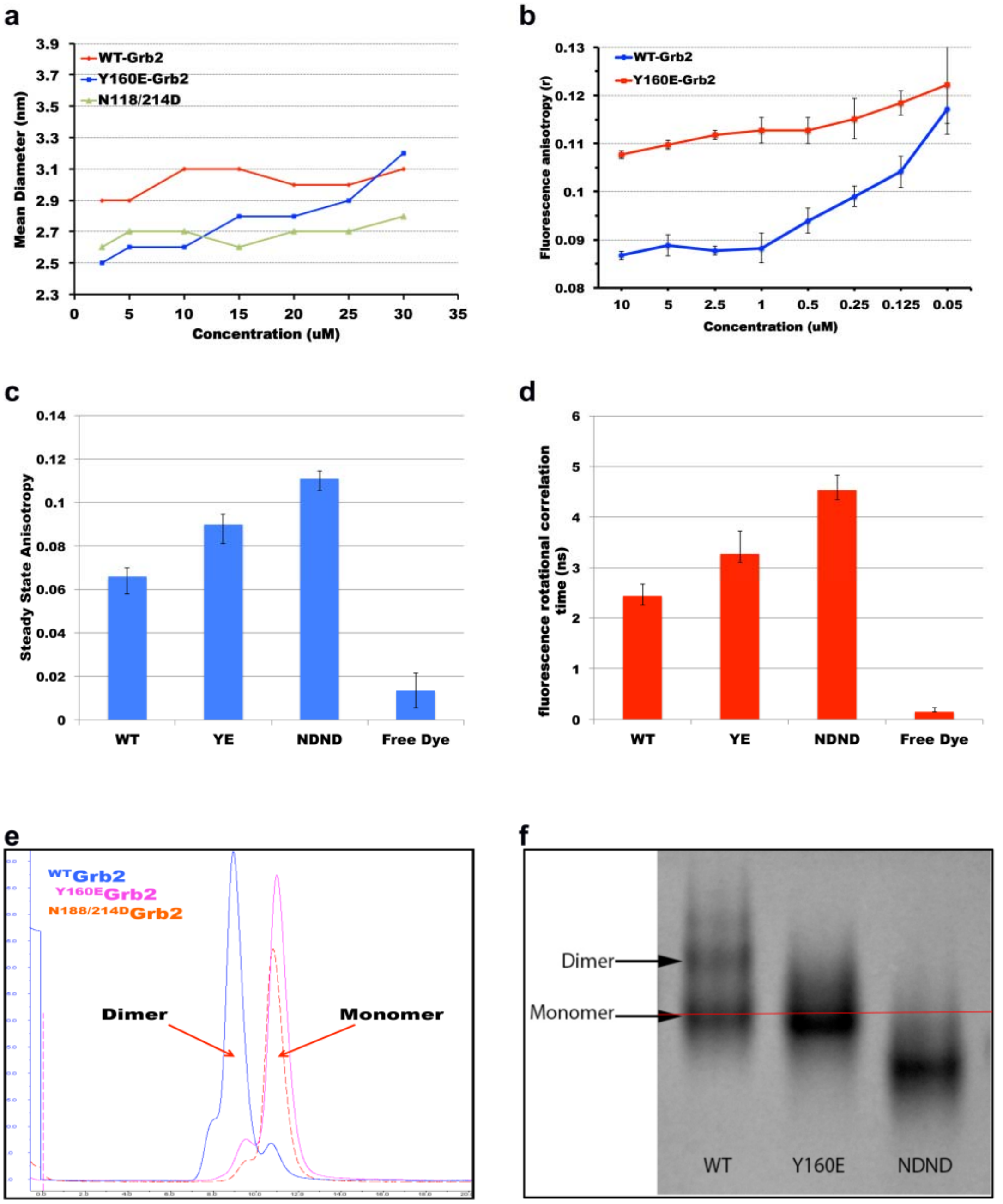

Supplementary Figure 2. Characterization of monomeric and dimeric states of Grb2. (a) Dynamic light scattering (DLS). Plots of hydrodynamic radius as a function of concentration for ^{WT}Grb2 (red), Y160E (blue) and N188/214D (green). Here DLS experiments show the mean diameter of wild type Grb2 is centered around 3.0 nanometer (nm) consistent with previously reported dimeric Grb2 radius (Lin et al., 2012). The mean diameter remains constant for ^{WT}Grb2 across the concentration range 2.5 μM to 30μM. Y160E has a lower mean diameter of approximately 2.6nm at lower concentrations. At higher protein concentrations the mean diameter reaches a value comparable to the wild type consistent with a potential for weak dimerization. The D188/214N mutant also shows a lower mean diameter like the Y160E, but it remains unchanged even at higher protein concentration. Note that the detection range of the instrument limits measurements to protein concentration above 2.5μM. **(b) Fluorescence anisotropy.** Wild type Grb2 exhibits an apparent dimerization constant comparable those seen previously and with MST.

However, the Y160E mutant displays no monomer-dimer transition, consistent with MST. Interestingly, the anisotropy (r) value of dimeric Grb2 (dGrb2) is smaller than those seen for monomeric-Grb2 (mGrb2). At lower concentration the ^{WT}Grb2 is monomeric and the anisotropy values are comparable to that of the Y160E mutant. Error bars show standard deviations, n=5. **(c and d) Time resolved fluorescence anisotropy.** The rotational correlation time for the ^{WT}Grb2 was significantly faster than those measured for Y160E and N188/214D. This suggests that dGrb2 adopts a compact ordered conformation while the mGrb2 is more extended/disordered resulting in slower motion and higher anisotropy value. Error bars show the accuracy of the measurements. **(e) Size exclusion chromatography.** ^{WT}Grb2 and the two mutants were separated by their size. 40μM of each protein were analyzed using Superdex75 gel filtration column at room temperature in standard PBS buffer. The results show that the wild type Grb2 is predominantly dimeric with small peak corresponding to monomer. Y160E and the N188/214D mutants are predominantly monomeric with small dimer peak. **(f) Native polyacrylamide gel electrophoresis (PAGE).** Native-PAGE shows that the migration pattern corresponds to a faster (monomeric) and a slower (dimeric) protein band. The mutants show a single species correspond to the monomeric ^{WT}Grb2. Note NDND denotes N188/214D with two additional negative charges resulting greater migration in the native-PAGE gel.

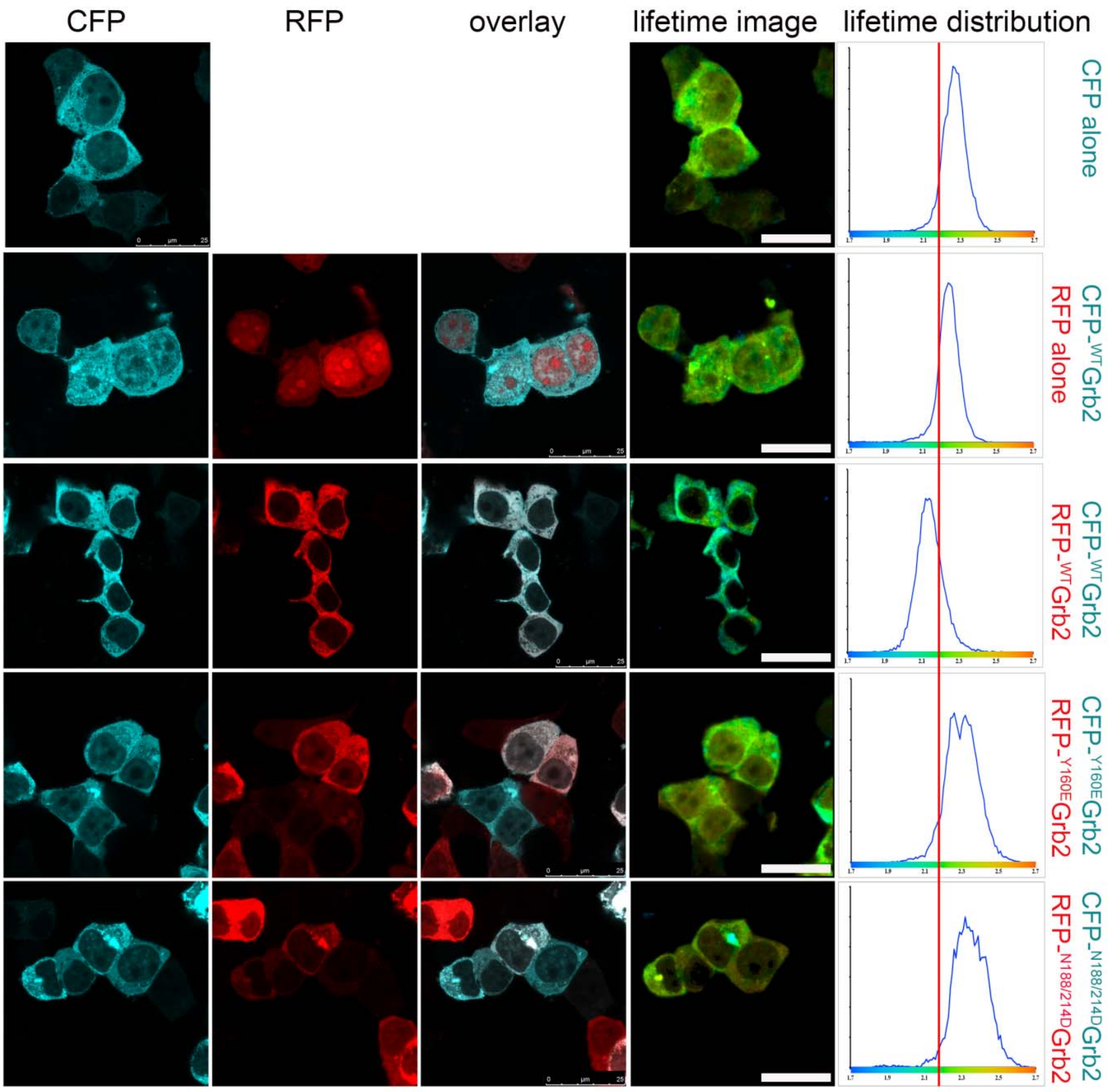

Supplementary Figure 3. Y160E and N188/214D mutants are monomeric inside cells. CFP tagged Grb2 (CFP-Grb2 (donor)) and RFP tagged Grb2 (RFP-Grb2, as (acceptor)) were co-transfected into HEK293T cells system and FLIM measurements were recorded and analyzed as previously reported[2-7]. Cells expressing both donor and acceptor Grb2 (CFP-^{WT}Grb2/RFP-^{WT}Grb2) show a reduction in CFP lifetime due to FRET. The lifetime plot (right column) shows significantly shorter average population lifetime compared to the controls visible as left-shifted peak relative to the arbitrarily drawn red line. This confirms Grb2 dimerization in a cellular context. For the Y160E and N188/214D mutants, despite considerable co-localization, no FRET was detected between protomers (see (CFP-^{Y160E}Grb2/RFP-^{Y160E}Grb2 and (CFP-$^{N188/214D}$Grb2/ RFP- $^{N188/214D}$Grb2). The data presented here are representative of two independent experiments and >20 cells from multiple field of view. Scale bar, 25μm.

Supplementary Figure 4. Monomer/dimer transition inhibited by the presence of Grb2-SH2 domain interacting phosphor-peptides but not in the presence of SOS peptides.

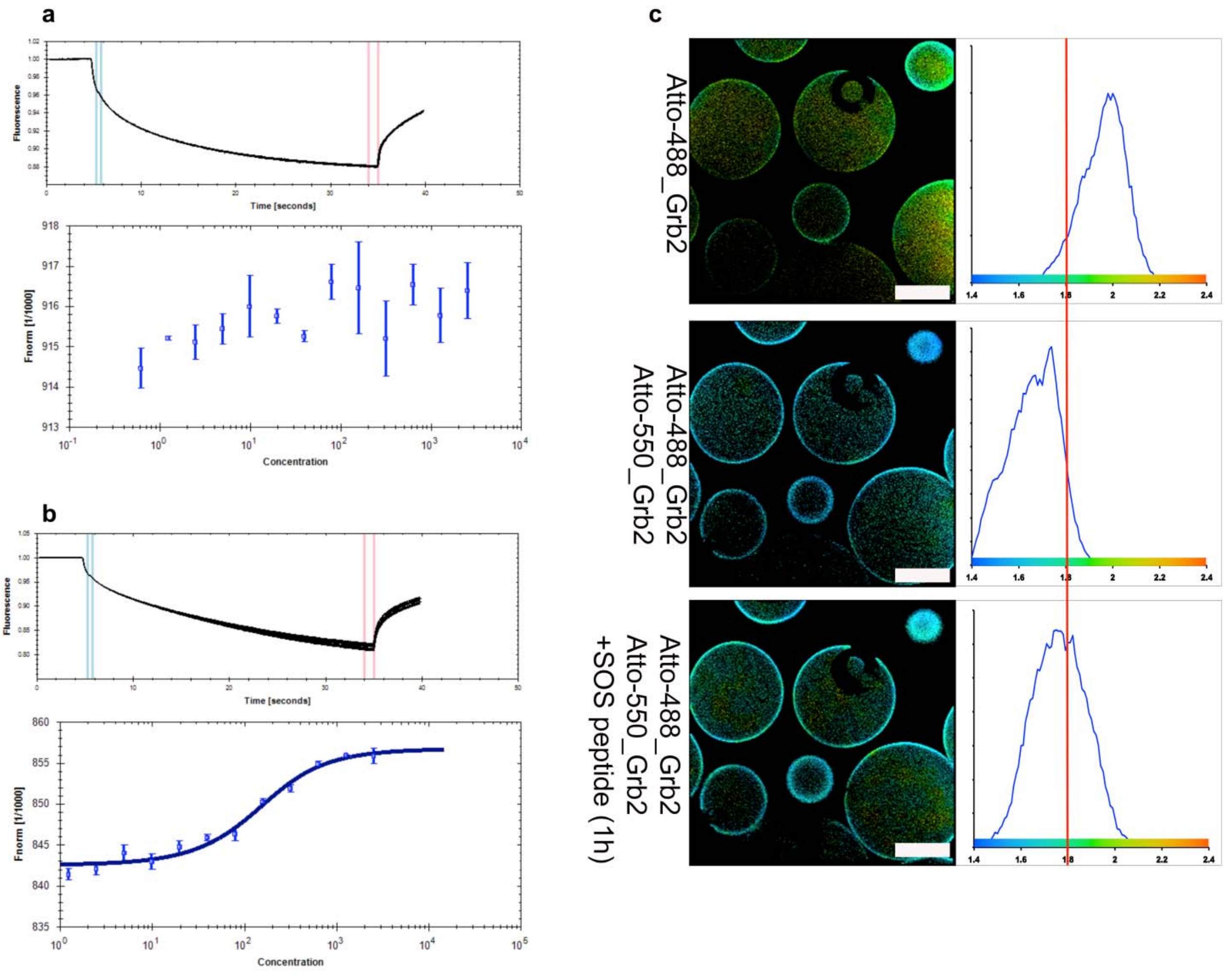

(a) Monomer-dimer equilibrium measured using MST. Unlabeled Grb2 protein (14.6 nM to 30 µM) was titrated into a fixed concentration of labeled Grb2 (200 nM) and 50 µM phosphoShc peptide. The top panel shows the raw data. The bottom panel shows the isotherm derived from the raw data. **(b)** MST experiments as above in the absence pShc peptide. Isotherm was fit to yield an apparent K_d, dimer (0.4 ± 0.20 µM). **(c)** SOS peptides do not disrupt FRET between Atto488-Grb2 and Atto550-Grb2. The experiment was carried out in PBS buffer pH7.5. The images represent distribution of the average fluorescence lifetime plotted to a false color map. The mean lifetime of Atto488-Grb2 in PBS is centered on ~1.9 ns. The addition of the acceptor Atto550-Grb2 leads to a left shift in the average lifetime, which is clear from the appearance of peak at ~1.6 ns as a result of FRET between Atto488-Grb2 and Atto550-Grb2. 50µM SOS peptide was added to the mixture and allowed to equilibrate for an hour. The lifetime measurements were retaken, however unlike the phospho-Shc peptide, SOS peptide does not induce dissociation of the Grb2 dimer. This is evident from the lack of change in the average lifetime between pre- and post-SOS peptide measurements. Scale bar, 50µm.

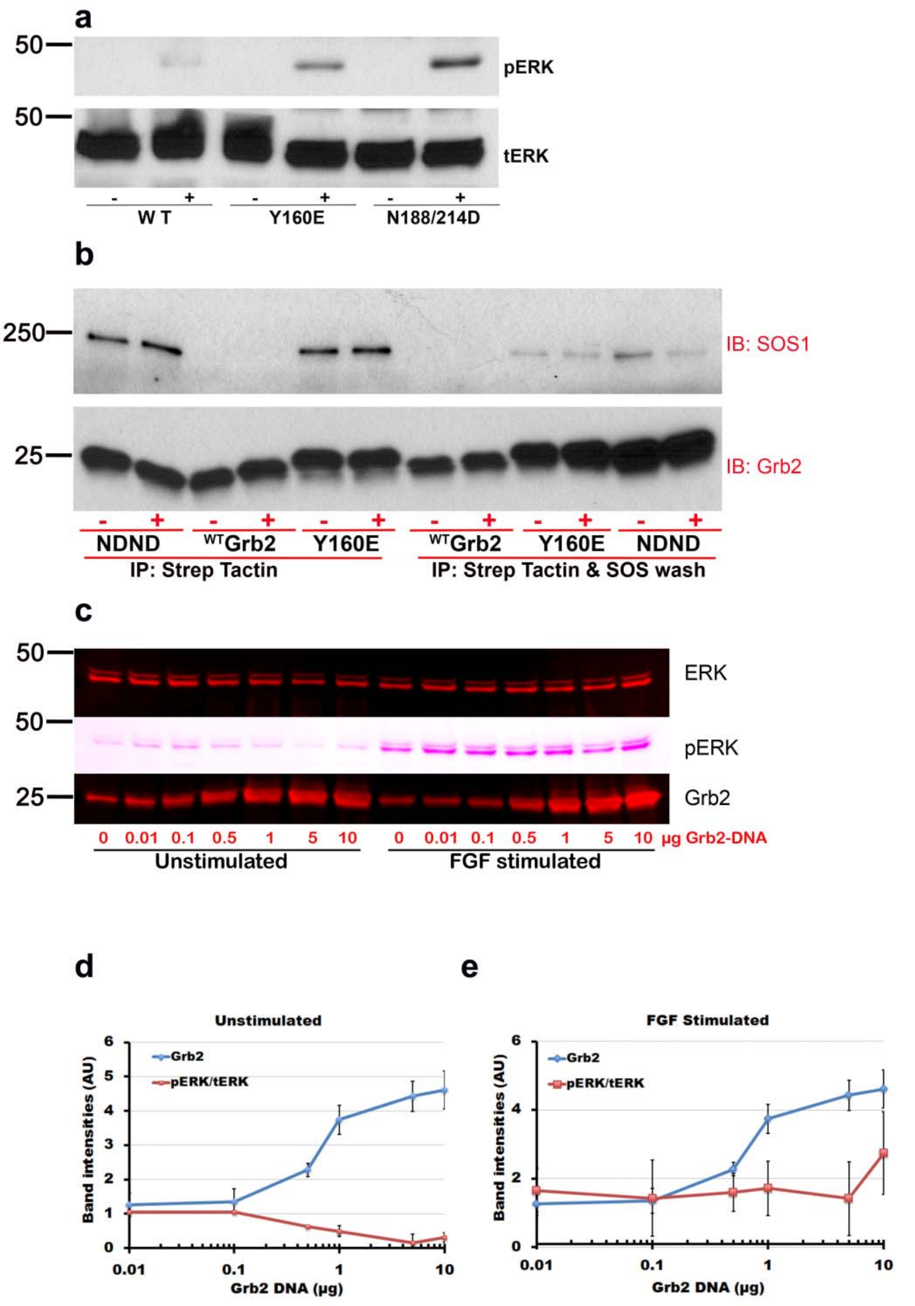

Supplementary Figure 5. Grb2 overexpression suppresses intracellular signaling. (a) HEK293T cells overexpressing FGFR2-GFP were stably transfected with either with strep-tagged ^{WT}Grb2, Y160E or N188/214D mutant, serum starved overnight and then stimulated with 50ng/ml FGF2 for 15 minutes. Cell lysates were analyzed for phospho-ERK, total ERK and Grb2 expression using specific antibody. The results show cell overexpressing wild type Grb2 has lower level of phospho-ERK activity than that of the dimerization defective mutants. **(b)** HEK293T cells overexpressing FGFR2-GFP and the indicated strep-tagged Grb2 were serum overnight and either left untreated or stimulated with FGF2 as above. 1000µg of total cell lysates were subjected to strep-tactin affinity purification. Following 4x lysis buffer wash each tubes were divided into two and subjected to either a lysis buffer alone or lysis buffer containing 10 µM SOS1 peptide wash (denoted as strep tactin & SOS wash). The resulting precipitants were analyzed for SOS and Grb2 binding using respective antibody. The results show both of the Grb2 dimerization defective mutants precipitate SOS while the ^{WT}Grb2 failed to do so. SOS peptide wash leads to a drastic reduction the level of precipitated SOS indicating direct complex formation by the mutants. Overnight serum starved samples are denoted as (-), 15min FGF2 stimulated samples denoted as (+), NDND denotes N188/214D. **(c)** Stable HEK293T cells overexpressing FGFR2-GFP were transiently transfected with increasing amount of strep-tagged wild type Grb2 plasmid DNA. Cells were serum starved overnight and then stimulated with 50ng/ml FGF9 for 15 minutes. Total cell-lysates were analyzed for phospho-ERK, total ERK and Grb2 expression. **(d)** Densitometry quantification graphs showing phospho-ERK level normalized against total ERK under non-stimulatory conditions. **(e)** Densitometry quantification for the level of phospho-ERK level normalized against total ERK following 15min FGF9 stimulation. The error bas represent standard deviation from two experiments.

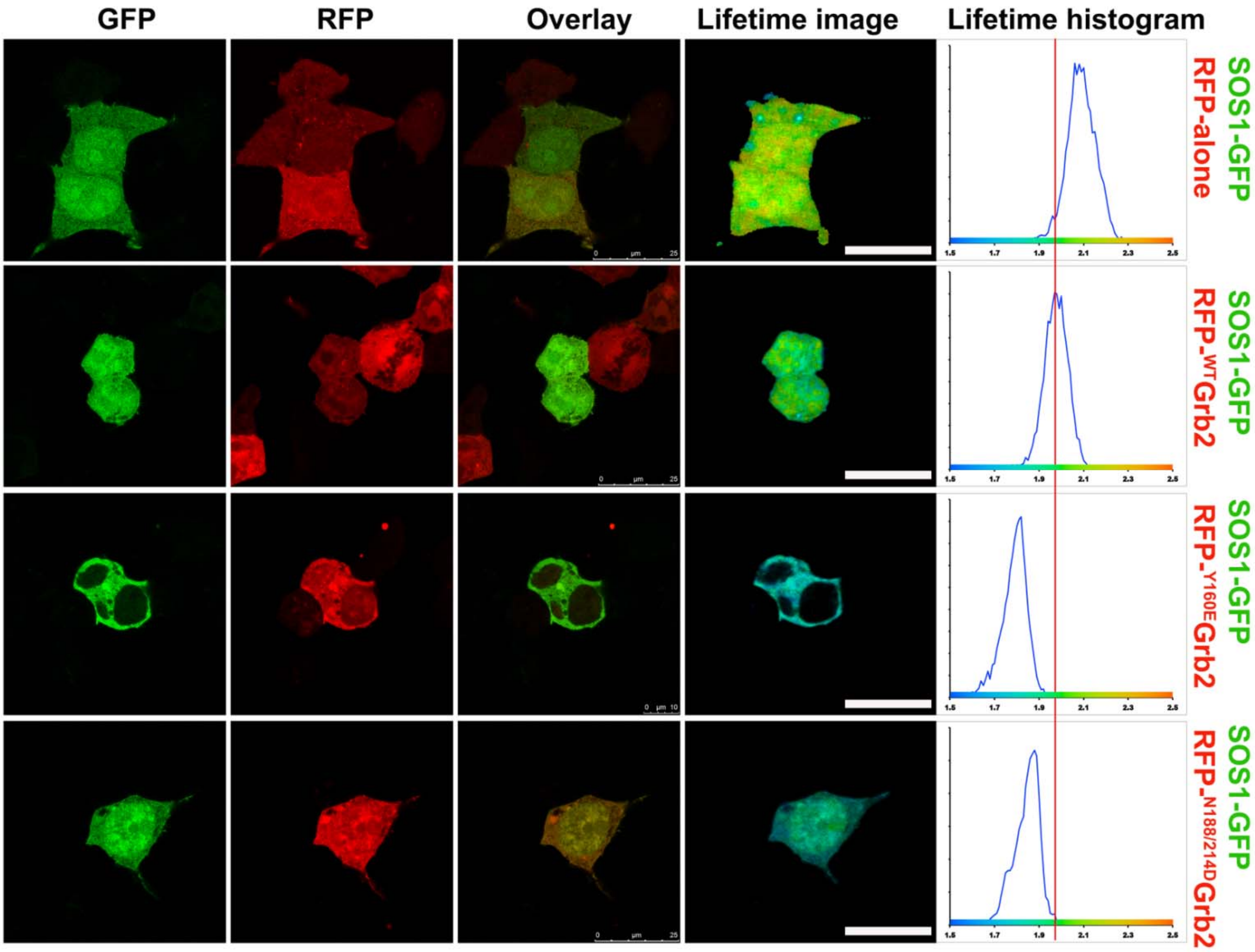

Supplementary Figure 6. Intracellular mGrb2 interaction with SOS1.

N-terminal GFP-tagged SOS1 (SOS1-GFP, as FRET donor) and RFP-tagged Grb2 (RFP-Grb2, as FRET acceptor) were co-transfected into HEK293T cells and confocal imaging and lifetime measurements were carried out. Cells co-transfected with SOS1-GFP and RFP vector alone were used as a control (see SOS1-GFP/RFP-alone). Both Y160E and N188/214D bind to SOS1. ^{WT}Grb2, which is overexpressed at concentrations where the population is likely to be dimeric[8] is unable to bind (left-shifted average lifetime in cells expressing the mutant Grb2 compared to the wild type). The data is represents >10 cells from two independent experiments. Scale bar, 25μm.

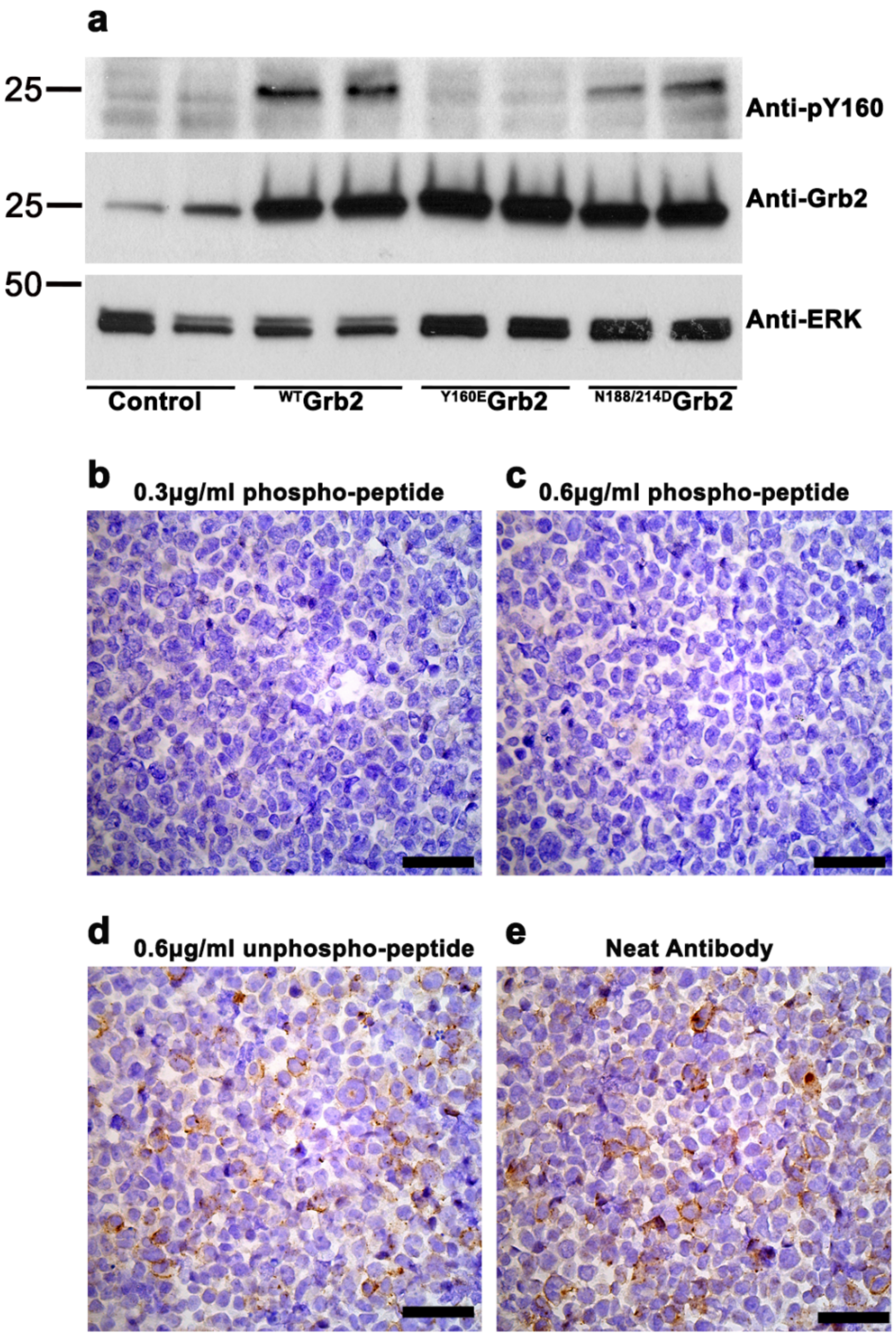

Supplementary Figure 7. Anti-pY160 antibody is suitable for IHC staining. We generated a site-specific antibody against the phosphorylated tyrosine Y160 of Grb2 (Anti-pY160). **(a)** The effectiveness of the antibody in western blotting was tested using cell-lysates of previously generated HEK293T cells overexpressing FGFR2-GFP[3] that were transfected with either Strep-tagged wild type, Y160E or N188/214D Grb2. Untransfected cells were used as a control. The pY160 antibody detected phosphorylated Grb2 in cells expressing the wild type and N188/214D but not in the Y160E showing that the pY160 antibody is specific for the phosphorylated tyrosine at position 160 of Grb2. Standard molecular weight markers are shown in kDa. **(b-e)** We also tested the pY160 antibody in immunohistochemistry (IHC) studies. HEK293T cells overexpressing FGFR2-GFP and Strep-tagged Grb2 were grown in 10cm dish, detached and pelleted. The cell-pellet was fixed with 4% paraformaldehyde in PBS and paraffin embedded, cell-slides were prepared. IHC staining was performed in the presence or absence of pY160 quenching peptides. **(b and c)** There is minimal or no staining of cells in the presence of the indicated concentration of quenching phospho-peptide. **(d)** There is moderate staining in the presence of un-phosphorylated quenching peptides. **(e)** There is marked or strong staining in the absence of quenching peptides (neat). The results here show that the pY160 antibody specifically detects Grb2 when phosphorylated at position 160. The bar shown on **b-e** corresponds to 50µm.

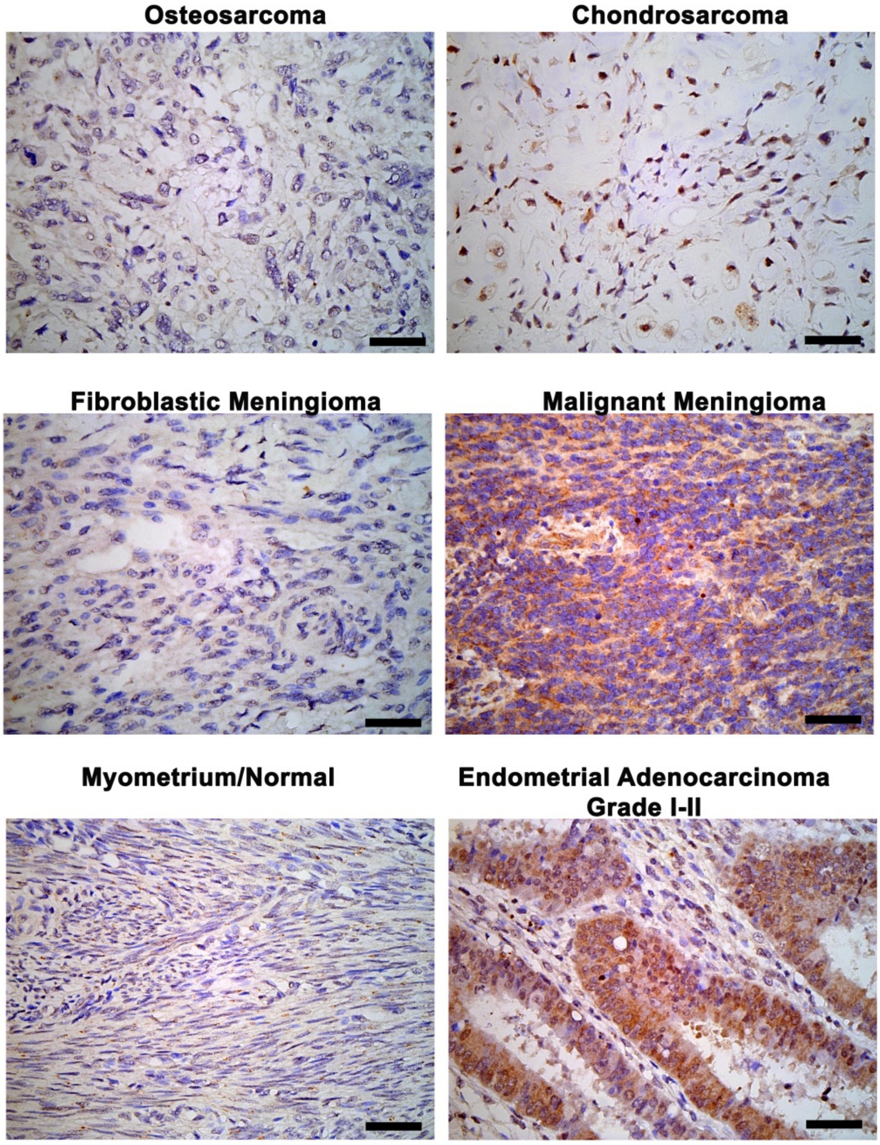

Supplementary Figure 8. Tumor microarray analysis showing high levels of pY160 staining associated with late stage malignant tumors.

IHC was performed on a multi-tumor tissue microarray containing 40 types tumors from 27 organs. In addition to colon and prostate adenocarcinoma, a significant rise in the level of Y160 phosphorylation was observed in chondrosarcoma, malignant meningioma and endometrial adenocarcinoma. The bar represents 50μm.

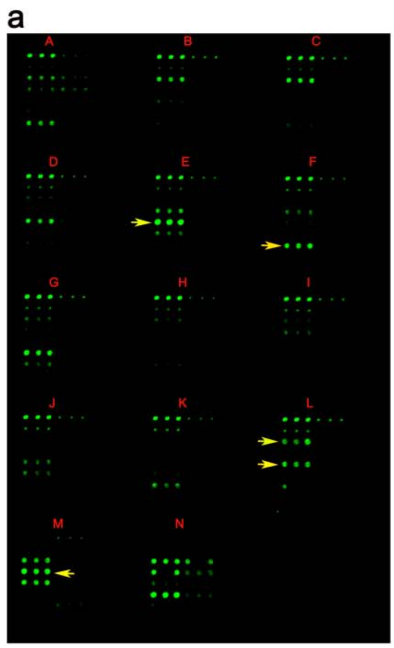

b

Grid position	p score	pY160 positive samples
A3	2	Ductal Carcinoma Grade 2 Stage IIa T2N0M0 Female Age 54
A7	3	Ductal Carcinoma Grade 2 Stage IV T4N2Mx Female Age 45
B3	3	Ductal Carcinoma Grade 2 Stage IIa T2N0M0 Female Age 42
C3	3	Ductal Carcinoma Grade 2 Stage IIb T3N0M0 Female Age 46
D5	3	Ductal Carcinoma Grade 2 Stage IV T4N0M0 Female Age 75
E4	2	Ductal Carcinoma Grade 3 Stage IIb T2N1M0 Female Age 39
E5	5	Ductal Carcinoma Grade 3 Stage II T2N1Mx Female Age 35
E6	1	Ductal Carcinoma Grade 3 Stage III T3N1bMx Female Age 73
F4	1	Ductal Carcinoma Grade 3 Stage IIIa T3N1M0 Female Age 49
F7	4	Ductal Carcinoma Grade 3 Stage II T2N1M0 Female Age 55
G6	4	Ductal Carcinoma Grade 3 Stage IV T4N2Mx Female Age 54
G7	1	Ductal Carcinoma Grade 3 Stage II T2N1M0 Female Age 43
I4	1	Ductal Carcinoma Grade 2 Stage IIb T2N1M0 Female Age 32
J5	2	Ductal Carcinoma Grade 1 Stage IIIa T3N1M0 Female Age 38
J6	2	Ductal Carcinoma Grade 1 Stage IIa T2N0M0 Female Age 60
K7	3	Metaplastic Carcinoma Grade 3 Stage IV T4N1bMx Female Age 46
L3	4	Human Cell Line A549 Lung Carcinoma
L5	4	Human Cell Line PC3 Prostate Adenocarcinoma
M3	3	Human Cell Line CRL-1976 Uterine Sarcoma
M4	5	Human Cell Line HT-29 Colon Adenocarcinoma
M5	4	Human Cell Line AGS Stomach Adenocarcinoma
N6	5	Human Cell Line Raji Burkitt Lymphoma

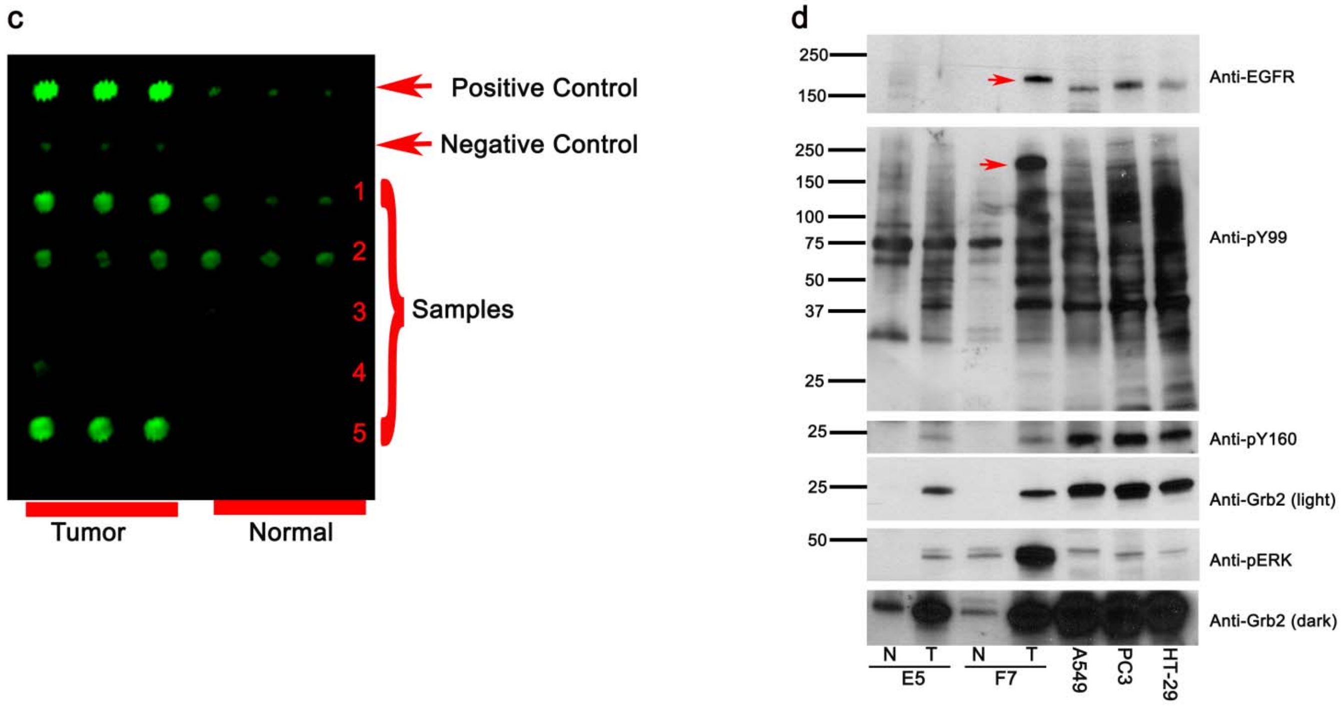

Supplementary Figure 9. Protein microarray analysis of 55 breast tumors with matched normal samples.
(a) Protein microarray slides showing specific tumors with Y160 phosphorylated Grb2. The grid numbers (A-N) are indicated in red. The yellow arrows indicate the samples selected for western blotting analysis. **(b)** A list breast tumor samples which tested positive for Y160 phosphorylation. The samples highlighted in green were selected for western blotting analysis and also indicated by the yellow arrows in (a). The p-scores represent an arbitrary number for the strength of the pY160 signal (with increasing signal strength marked from 0 to 5). **(c)** A guide to the array grid and sample locations. For more details see the PDF document at: http://www.proteinbiotechnologies.com/products/protein_microarrays/human_cancer_protein_microarray_PMA2-001-L.html. **(d)** Western blot analysis of tumor (denoted on the blot as T) and the matched normal (denoted on the blot as N) tissues from the protein microarray with high levels of Y160 phosphorylation. Prostate, colon and lung cancer cell lines that tested positive for Y160 phosphorylation were also analyzed in the

same blot. Cell lysates were purchased and analyzed with anti-phosphotyrosine (Anti-pY99), anti-pY160, anti-pERK and anti-EGFR antibody. The red arrows indicate phosphorylated EGFR in the pY99 immunoblot and the corresponding increased receptor band in the anti-EGFR immunoblot. Here the results show both tumor samples have high levels of tyrosine kinase activity as seen with anti-pY99 immunoblot. It is also evident that increased Grb2 expression is coupled with increased Grb2 tyrosine phosphorylation on Y160, which correlates with increased MAP kinase activity. However, the extent of Grb2 pY160 phosphorylation in cultured cell lines was much higher than that of the primary tumours, which did not correlate with higher pERK. This could be due to specific environmental effects. *In vivo* solid tumors are exposed to gradients of chemical and biological signals which can exert both stimulatory and inhibitory effects on tumor progression. Unlike the complex tumour microenvironment, cultured cells are exposed to uniform oxygen and nutrients. Therefore, the discrepancies are most likely related to the growth conditions and specific environment of cells. This is further exemplified here by the observation that not all breast tumour samples exhibited higher pY160 phosphorylation.

Supplementary Figure 10. Un-cropped western blots. The blue rectangles indicate the cropped area used in respective figures.

Related to figure 4a

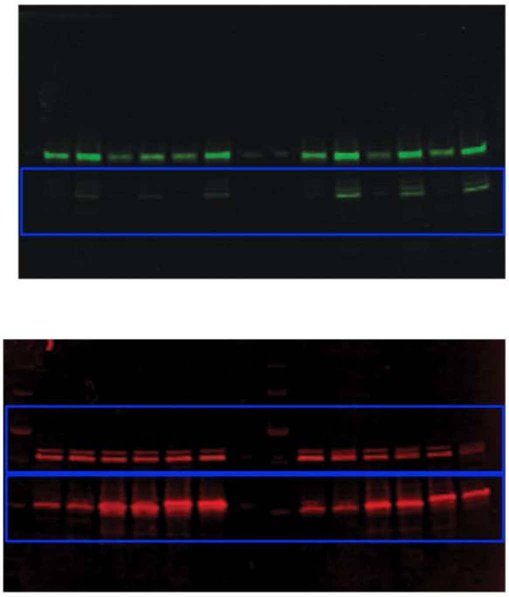

Related to figure 4b

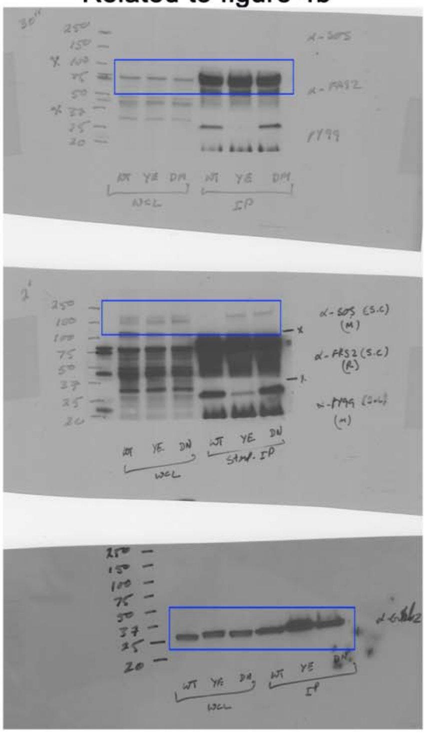

Related to figure S5a

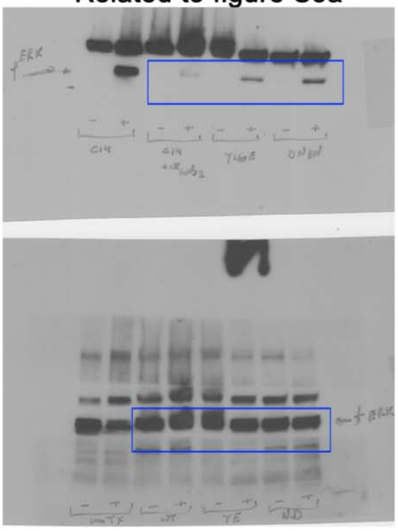

Related to figure S5c

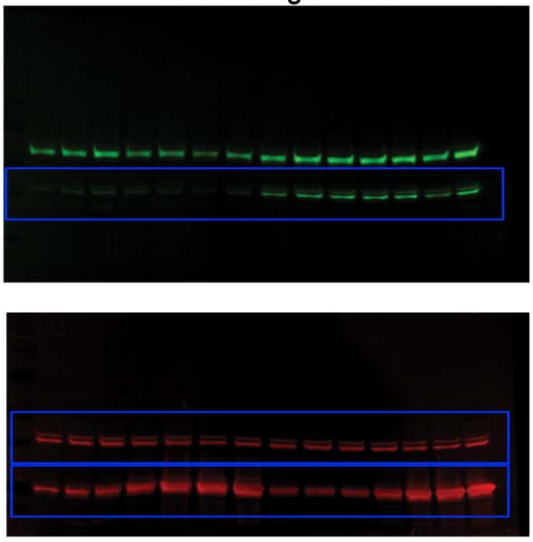

Related to figure S5b

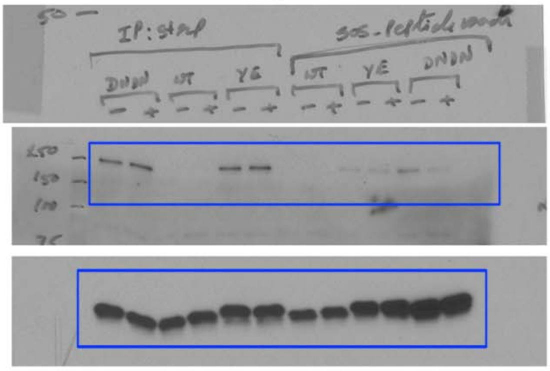

Supplementary Note 1

Dynamic light scattering (DLS). DLS experiments were performed at 20°C in a Wyatt Technologies DynaPro Titan instrument. Experiments were carried out in standard PBS buffer. Grb2 samples were first filtered and then centrifuged at 16,100 × g for 10min at 20°C to remove residual aggregates. DLS data were collected and analyzed using DYNAMICS V6.7.6 software for the DynaPro Titan instrument (Wyatt Technology Corporation).

Fluorescence anisotropy. In order to further confirm the monomeric status of the Y160E we performed fluorescence anisotropy experiments. Our rational is that a dimeric Grb2 would be a much larger than the monomeric protein resulting in a substantial change in anisotropy. Fluorescent anisotropy experiments were performed on a Horiba Jobin Yvon Fluorolog 3 in 4 mm semi-quartz cuvettes from Starna Cells. Excitation of the internal tryptophan was set at 290 nm with emission measured at 350 nm. The integration time for each measurement was set at 1 second and 5 accumulations for each concentration were taken and then averaged. The anisotropy was determined by instrument software using the formula:

$$r = \frac{I_{VV} - (G \times I_{VH})}{I_{VV} + (G \times 2 \times I_{VH})}$$

Intensity (I) with subscripts refers to orientation of the excitation and emission polarizer respectively. G is the G-factor and is calibrated for the wavelengths used and fixed for all measurements.

Time-resolved fluorescence anisotropy. Purified proteins were N-terminally labeled with Atto488 dye (Atto-Tec) according to the manufacturer's instruction. The fluorescence anisotropy and rotational correlation time was measured using FT300 spectrometer (PicoQuant) at 25°C. Samples were excited at 465nm and emission was detected at 520nm with a hybrid PMT detector. The anisotropy and rotational correlation time was determined by the instrument software. Larger molecules generally have a higher anisotropy value then a smaller counterpart. Viscosity, molecular weight, hydration, molecular shape and association with other molecules can influence anisotropy decay analysis. Here the molecular shape could have an influence on the observed inverse correlation between the larger dGrb2 and the smaller mGrb2. We investigated this using time resolved fluorescence anisotropy to determine the rotational correlation time between the wild type and mutant Grb2. The rationale is that if the conformation of mGrb2 is extended/disordered in solution which affects its rotation and anisotropy. The observed anisotropy and correlation time is higher in the monomer than in the dimer. This is due to homo-FRET in the dimers, which does not occur in the monomers. Homo-FRET is not a concentration-dependent effect, but is due to the short distance between the Atto fluorescent dyes in the context of a dimer. The correlation time observed does not strictly represent the movement of the protein. The anisotropy decay of a dye covalently attached to a protein is dominated by the local rotational freedom of the dye and by the segmental mobility of the protein backbone where the dye is attached. These processes are much faster than the rotation of the protein itself. The obtained phi values are interpreted as the average correlation time of the depolarization by these two faster processes, along with depolarization due to homo-FRET in the case of the dimer. Note that the "free dye" here represents Coumarin6, correlation time and steady-state anisotropy are representative of free dyes in fluid solvents at room temperature

1. Jerabek-Willemsen, M., Wienken, C.J., Braun, D., Baaske, P., and Duhr, S. (2011). Molecular interaction studies using microscale thermophoresis. Assay Drug Dev. Technol. *9*, 342-353.

2 Suhling, K., Siegel, J., Phillips, D., French, P.M., Leveque-Fort, S., Webb, S.E., and Davis, D.M. (2002). Imaging the environment of green fluorescent protein. Biophys. J. *83*, 3589-3595.
3 Ahmed, Z., Schuller, A.C., Suhling, K., Tregidgo, C., and Ladbury, J.E. (2008). Extracellular point mutations in FGFR2 elicit unexpected changes in intracellular signalling. Biochem J. *413*, 37-49.
4 Schuller, A.C., Ahmed, Z., Levitt, J.A., Suen, K.M., Suhling, K., and Ladbury, J.E. (2008). Indirect recruitment of the signalling adaptor Shc to the fibroblast growth factor receptor 2 (FGFR2). Biochem. J. *416*, 189-199.
5 George, R., Chan, H.L., Ahmed, Z., Suen, K.M., Stevens, C.N., Levitt, J.A., Suhling, K., Timms, J., and Ladbury, J.E. (2009). A complex of Shc and Ran-GTPase localises to the cell nucleus. Cell. Mol. Life Sci. *66*, 711-720.
6 Ahmed, Z., George, R., Lin, C.C., Suen, K.M., Levitt, J.A., Suhling, K., and Ladbury, J.E. (2010). Direct binding of Grb2 SH3 domain to FGFR2 regulates SHP2 function. Cell. Signal. *22*, 23-33.
7 Ahmed, Z., Lin, C.C., Suen, K.M., Melo, F.A., Levitt, J.A., Suhling, K., and Ladbury, J.E. (2013). Grb2 controls phosphorylation of FGFR2 by inhibiting receptor kinase and Shp2 phosphatase activity. J. Cell Biol. *200*, 493-504.
8 Lin, C.C., Melo, F.A., Ghosh, R., Suen, K.M., Stagg, L.J., Kirkpatrick, J., Arold, S.T., Ahmed, Z., and Ladbury, J.E. (2012). Inhibition of basal FGF receptor signaling by dimeric Grb2. Cell *149*, 1514-1524.
